# Identification of ungulates used in a traditional Chinese medicine with DNA barcoding technology

**DOI:** 10.1002/ece3.1457

**Published:** 2015-04-08

**Authors:** Jing Chen, Zhigang Jiang, Chunlin Li, Xiaoge Ping, Shaopeng Cui, Songhua Tang, Hongjun Chu, Binwan Liu

**Affiliations:** 1 Key Laboratory of Animal Ecology and Conservation Biology, Institute of Zoology, Chinese Academy of SciencesChaoyang District, No. 1 Beichen West Road, Beijing, 100101, China; 2 University of Chinese Academy of SciencesNo. 19 (A) Yuquan Road, Beijing, 100049, China; 3 Endangered Species Scientific Commission of the People’s Republic of ChinaChaoyang District, No. 1 Beichen West Road, Beijing, 100101, China; 4 School of Resources and Environmental Engineering, Anhui UniversityNo. 111 Jiu Long Road, Hefei, 230601, Anhui Province, China; 5 Wildlife Conservation Office of Altay Prefecture836500, Altay, Xinjiang, China; 6 College of Wildlife Resources, Northeast Forestry University, No. 26 Hexing Road Xiangfang DistrictHarbin, 150040, Heilongjiang Province, China

**Keywords:** Conservation genetics, DNA barcode, DNA identification, illegal trade, traditional Chinese medicines, wildlife forensics

## Abstract

Horns of Saiga antelope (*Saiga tatarica*) have always been an ingredient of “Lingyangjiao”, a traditional Chinese medicine (TCM). Persistent hunting for Saiga antelope has already threatened the survival of critical endangered populations in wild. To control the growing pressure, CITES and Chinese government have legislated for monitoring the trade of Saiga horns. However, similar ungulate horns are difficult to identify by their morphological characteristics, which has impeded the law enforcement. Besides Saiga antelope, other seven ungulate species which have similar horns are also sold and marked as “Lingyangjiao” in TCM markets to offset shortage of Saiga antelope horns. Such species are *Gazella subgutturosa*, *Pantholops hodgsonii*, *Procapra picticaudata*, *Procapra gutturosa*, *Procapra przewalskii*, *Capra hircus,* and *Ovis aries*. Our study aimed at implementing DNA barcoding technology to diagnose Saiga horns and the substitutes. We successfully extracted genomic DNA from horn samples. We recovered COI sequences of 644 bp with specific primers and 349 bp with nested PCR primers designed for degraded horn samples. The mean interspecific genetic distance of data set of the 644-bp full barcodes and the 349-bp mini-barcodes was 14.96% and 15.38%, respectively, and the mean intraspecific distance was 0.24% and 0.20%, respectively. Each species formed independent clades in neighbor-joining (NJ) phylogenetic tree of the two data sets with >99% supporting values, except *P. gutturosa* and *P. przewalskii*. The deep genetic distances gap and clear species clades in NJ tree of either full barcodes or mini-barcodes suggest that barcoding technology is an effective tool to diagnose Saiga horns and their substitutes. Barcoding diagnosis protocol developed here will simplify diagnosis of “Lingyangjiao” species and will facilitate conservation of endangered ungulates involved in TCM “Lingyangjiao” markets, especially the Saiga antelope.

## Introduction

“Lingyangjiao”, a traditional Chinese medicine (TCM), has been used for almost 2,000 years in Chinese communities (Chan et al. 1995). It especially refers to the horns of Saiga antelope (*Saiga tatarica*), a migratory ungulate living in the steppe and semi-desert regions of central Asia and south-eastern Europe (Sokolov 1974). As one of the major consumers of “Lingyangjiao”, China imported 34,851 kg of Saiga horns during period of 1995-2004 according to trade databases of CITES and TRAFFIC (Chan et al. 1995; Li et al. 2007; Von Meibom et al. 2007, 2010). During the same period, 4000 to 5000 kg of Saiga horns was confiscated in 27 cases of smuggling into China (Von Meibom et al. 2007). From 2005 to June 2012, another 19 cases of illegal trade were reported and nearly 16,762 horns were confiscated. As only male Saiga antelope bear horns, the selected hunting for males has resulted in not only a direct decline of population sizes but also reproductive collapse (Milner-Gulland et al. 2001, 2003; Kuehl et al. 2009). To bring this species back from the brink of extinction, Saiga antelope was listed in Appendix II of CITES in 1995 and listed as First Category of National Key Protected Wild Animals of China in 1989 (Jiang et al. 1996; Milner-Gulland et al. 2001). Markets of Saiga horns have been closely monitored, and any trade of Saiga horns without permit issued by national wildlife management authority or national CITES authority has been forbidden by national law in China.

Supply of Saiga horns has been decreasing as decline of Saiga population and rigorous control on Saiga horn trade; however, demand of “Lingyangjiao” is still booming in TCM market. To compensate the shortage of Saiga horns, horns from some other species are used as substitutes in underground markets even legal markets. In 2012, DNA of domestic sheep (*Ovis aries*) and domestic goat (*Capra hircus*) was detected in bottles of TCM “Lingyangjiao” powder, which was claimed to be 100% of Saiga horns (Coghlan et al. 2012). Besides sheep and goat, horns of goitered gazelle (*Gazella subgutturosa*), Tibetan antelope (*Pantholops hodgsonii*), Tibetan gazelle (*Procapra picticaudata*), Mongolian gazelle (*Procapra gutturosa*), and Przewalski’s gazelle (*Procapra przewalskii*) are also marked and sold as “Lingyangjiao” in TCM markets without declaration of the real ingredients (Liu 1982; Chan et al. 1995). These wild ungulates are all listed as national key protected wild animals of whose trade is also forbidden (Table1) (Jiang et al. 1996). We named all these species whose horns were sold as “Lingyangjiao,” the “Lingyangjiao” species in this study. Horns of these species often have similar morphological characteristics and are hard to distinguish from each other especially when they are sold in slices, lumps, or powders. Disorder of “Lingyangjiao” markets impedes not only control of Saiga horns trade but also conservation of Saiga antelope and the other endangered ungulates. Therefore, an effective and convenient method to identify the ingredients of “Lingyangjiao” is imperatively required.

**Table 1:** Summary of the specimens of eight species in “Lingyangjiao” markets

Species	Common name	Subfamily	IUCN status^1^	Legal status in China^2^	Number of samples \sequences	Types of samples
*Procapra picticaudata*	Tibetan gazelle	Antilopinae	Near Threatened	II	12\12	Muscles and skins
*Procapra przewalskii*	Przewalski’s gazelle	Antilopinae	Endangered	I	12\14	Skins and horn
*Procapra gutturosa*	Mongolia gazelle	Antilopinae	Least Concern	II	10\11	Muscles and horn
*Gazella subgutturosa*	Goitered gazelle	Antilopinae	Vulnerable	II	9\9	Muscles
*Saiga tatarica*	Saiga antelope	Antilopinae	Critically Endangered	I	6\7	Skins and horns
*Pantholops hodgsonii*	Tibetan antelope	Antilopinae	Endangered	I	3\5	Skins
*Capra hircus*	Goat	Antilopinae	–	–	4\7	Horns
*Ovis aries*	Sheep	Antilopinae	–	–	0\4	–

1 IUCN Red List of Threatened Species, Version 2014.

2 I First Category of National Key Protected Wild Animals; II Second Category of National Key Protected Wild Animals.

Proved to be qualified representative of mitochondrial genes, cytochrome c oxidase subunit 1 (COI) gene is a promising genetic marker in wildlife forensics, especially after proposed as standard DNA barcoding sequence in 2003 (Hebert et al. 2003a,b; Dawnay et al. 2007; Eaton et al. 2010; Dalton and Kotze 2011; Luo et al. 2011). The DNA barcoding system tries to provide an adequate and authoritative reference sequence library, which is indispensable information in wildlife identification (Dawnay et al. 2007; Ratnasingham and Hebert 2007; Wilson-Wilde et al. 2010). With an increasing number of projects launched to barcode numerous eukaryotic species, government could take full advantage of the barcoding system in wildlife forensics and conservation (Frezal and Leblois 2008; Ogden et al. 2010; Gathier et al. 2013). As to the case of identifying “Lingyangjiao” species, if all the candidate species were already barcoded, only the same sequences of tested samples were required and efforts of obtaining reference sequences could be saved. Nevertheless, before the diagnostic potentiality of barcoding technology is put into practice, a comprehensive and authoritative reference sequences library still needs to be constructed through the full cooperation and collaboration of the taxonomists, biologists, and conservationists.

In this study, we used DNA barcoding technology to identify “Lingyangjiao” species: *S. tatarica*, *G. subgutturosa* and *P. hodgsonii*, *P. picticaudata*, *P. gutturosa*, *P. przewalskii*, *C. hircus,* and *O. aries*. We attempted to extract genomic DNA and recover COI gene from horn samples to determine whether the horns could be used in molecular forensics. We expected to build an effective protocol for “Lingyangjiao” samples and provide a series of reference sequences for quick diagnosis.

## Materials and Methods

### Sample collection

**Figure 1:**
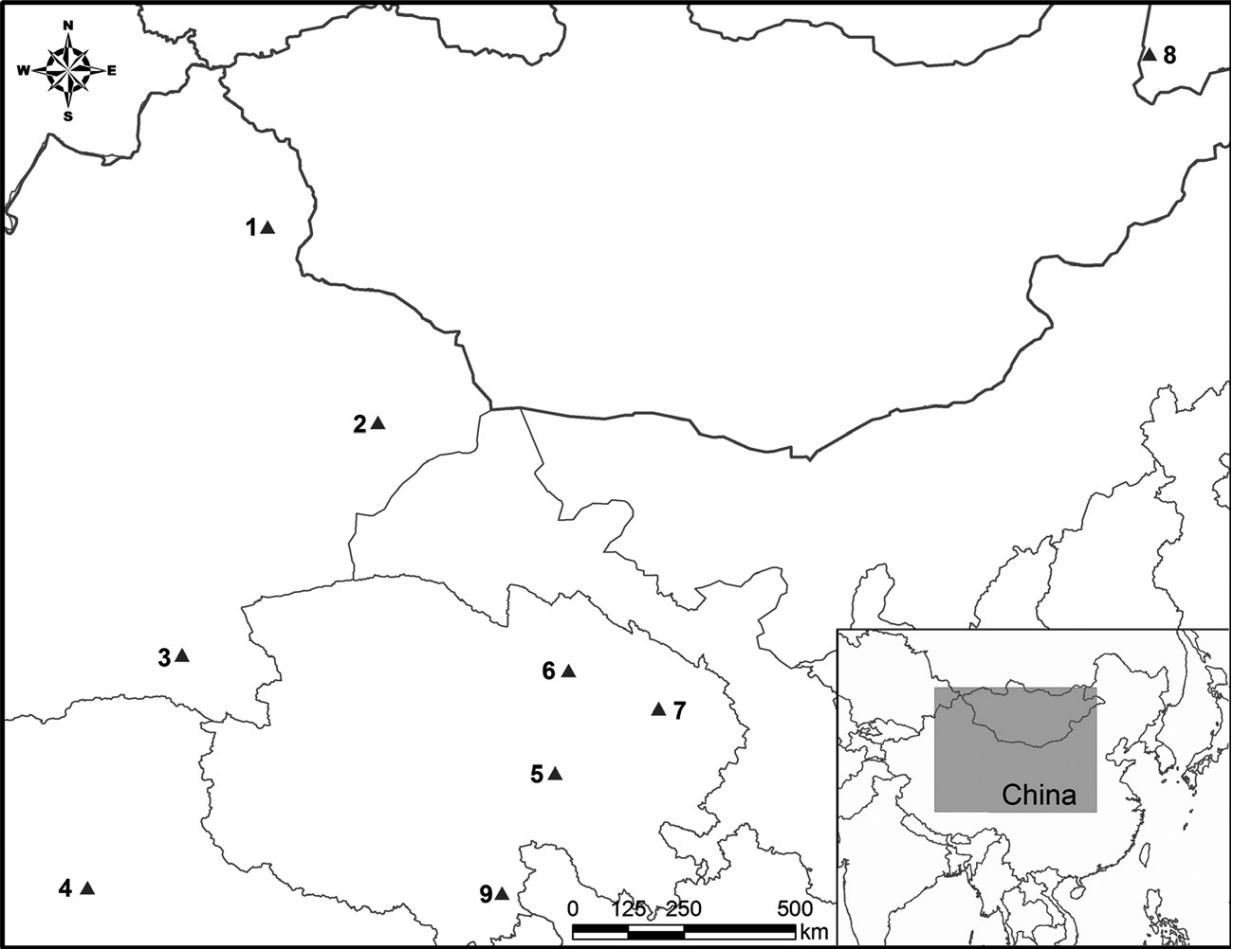
Locations of samples (black triangles) collected for barcoding of species involved in TCM “Lingyangjiao” markets. 1: *Gazella subgutturosa*; 2: *Capra hircus*; 3, 4, and 5: *Procapra picticaudata*; 6 and 7: *Procapra przewalskii;* 8: *Procapra gutturosa;* 9: *Pantholops hodgsonii*.

Fifty-four samples of *S. tatarica* and the other “Lingyangjiao” species were collected (Table1 and Fig.1). In detail, one skin sample of Saiga antelope was sampled from a specimen preserved in the Museum of Xinjiang Institute of Ecology and Geography, Chinese Academy of Sciences. A whole Saiga horn sample was provided by Forestry Bureau of Hebei Province and identified by Zhigang Jiang. Other four samples of old Saiga horns, stored for a long time and processed into lump, slice, and two powders, respectively, were provided by a TCM producer. A mitochondrial genomic sequence of Saiga antelope (JN632700) from GenBank (http://www.ncbi.nlm.nih.gov/genbank/) was also employed in this study. Two muscle samples of *P. przewalskii* were from Museum of Institute of Zoology, Chinese Academy of Sciences. The other nine skins which were also used in study of Yang et al. (2011) and one horn of *P. przewalskii* were collected from the carcasses found in distribution range of the species in Qinghai Province. Furthermore, two sequences of mitochondrial genome of *P. przewalskii* were employed (NC_014875, GU386355). Twelve muscle and skin samples of *P. picticaudata* were collected from local hunters in Qinghai, Tibet, and Xinjiang which were also used in study of Zhang and Jiang (2006). Nine muscle samples and a horn of *P. gutturosa* were provided by A Day Hasha Te Manzhouli Customs, Inner Mongolia, which were cut from bodies confiscated as smuggled goods. A mitochondrial genomic sequence of *P. gutturosa* was also employed (JN632689). Two samples of *P. hodgsonii* were provided by A-erh-chin Mountain Natural Reserve, and the other three sequences were retrieved from Genbank (DQ191826, HQ269460-HQ269461). Three horn lumps of domestic goat (*C. hircus*) were collected from local grassland in Shangshang County, Hami District, Xinjiang. The other sequences of domestic goat (HQ269452, HQ269437-HQ269439) and sheep (NC_001941, AF010406, FJ958344, and FJ958345) were retrieved from GenBank. All the samples were preserved in 95% ethanol or froze at −20°C. The horn samples used here were taken as representative samples of “Lingyangjiao” sold in TCM markets to evaluate their usability.

### DNA extraction, amplification, and sequencing

For the whole horns, middle layer between the bone core and outer sheath was sampled and pulverized into powder in the liquid nitrogen before digestion. The middle layer was almost as soft as skins and was supposed to have more cells then the other part of horns. The lump and slice horns were cut into smaller pieces by a power saw and pulverized into powder. Genomic DNA was extracted from all the muscle, skin, or horn samples using Universal Genomic DNA Extraction Kit Ver.3.0 (TaKaRa, Otsu, Shiga, Japan). Extraction processes followed the manufacturer’s instructions of the kit except that digestion was prolonged for two extra hours. Yields of genomic DNA were checked by agarose gel electrophoresis, and the final concentrations were measured by NanoDrop 2000 Spectrophotometer (Thermo Fisher Scientific, Waltham, Massachusetts, USA).

We designed a specific primer pair, 5COIf (5′-TGAGCCGGCATAGTAGGAAC-3′) and 5COIr (5′- CCTGAGTAGTAGGTGACAATGTG-3′), according to mitochondrial genome of *S. tatarica*, *P. przewalskii*, *P. hodgsonii*, *O. aries*, and *C. hircus* with Primer Premier 6.0 (Premier Biosoft International, Palo Alto, California, USA). The primer set targeted 714-bp COI sequences from 5391 bp to 6105 bp in *P. przewalskii* mitochondrial genome. However, the primer failed at the two powder samples of Saiga horns. We designed a nested PCR system in case of any other highly degraded samples collected from TCM markets. The outer primer was 5COI, and the inner primer was saigaCOIf (5′-GTAGTCGTAACCGCACAT-3′) and saigaCOIr (5′-GTAGGAGGACAGCCGTAAT-3′).

Amplification conditions of the two primer sets were optimized using gradient PCR. Reaction of 5COI was performed in a 40 *μ*L volume containing 1 × PCR buffer (TaKaRa, Otsu, Shiga, Japan), 2.0 mmol/L MgCl_2_, 0.2 mmol/L each dNTP, 0.5 *μ*mol/L each primer, 1 units Ex Taq DNA polymerase (TaKaRa, Otsu, Shiga, Japan), and 2.0 *μ*L genomic DNA. Amplification cycles were carried out on a Veriti 96-well Thermal Cycler (Applied Biosystems, Foster City, California, USA). The PCR thermal cycling profile of primer 5COI was 7 min at 94°C for initial polymerase activation, followed by 35 cycles of 30 sec at 94°C, 45 sec at 52°C, and 1 min at 72°C, with a final extension for 10 min at 72°C. For the two powder horn samples with low genomic DNA concentrations, two rounds of nested PCR were performed. Firstly, primer set 5COI was used as outer primer, and the amplification was performed identically to the former single-step PCR except that the cycles numbers was 20. Two microliters of products of the first reaction were used as templates for the second round with primer saiga COI, and the PCR conditions were the same as the former, except 54°C annealing temperature. Amplification success of the PCRs was checked by electrophoresis on 1.5% agarose gel. PCR products with robust and specific bands were sequenced with an ABI PRISM 3730XL DNA sequencer (Applied Biosystems, Foster City, California, USA). COI sequences were deposited in GenBank under Accession Numbers KC678998-KC679051.

### Data analysis

Chromatograms of COI sequences were checked by eyes with BioEdit Sequence Alignment Editor Version 7.0.5.3 (Ibis Bioscience, Carlsbad, California, USA), and ambiguous bases were trimmed (Hall 1999). Alignment was implemented in ClustalX 1.8.1 (Thompson et al. 2002). MEGA 4.0, which was proposed in barcoding systems, was employed to construct neighbor-joining (NJ) phylogenetic tree and calculate nucleotide sequence divergences based on Kimura 2-parameter model (Hebert et al. 2003b; Tamura et al. 2007). Missing data were completely deleted. Bootstrap values for the internal topology were estimated by 1000 replicates. To compare the ability of assigning a specimen to correct species between full-length barcodes and mini-barcodes, data set of 349-bp fragments was also used to construct NJ tree and calculate genetic distances.

## Results and Discussion

Genomic DNA of “Lingyangjiao” from TCM markets was likely to be degraded because they were sometimes processed into slice, block, or powder before sale and stored at room temperature and high humidity for a long period. Besides, cells in horns were surrounded by keratinized tissues, which were inherently difficult to digest. In this study, the whole horn samples of *S. tatarica*, *P. przewalskii*, *P. gutturosa* that were fresh and well-preserved yielded 2–10 *μ*g total DNA. DNA yields of old or processed horn samples of *S. tatarica* and *C. hircus* were so poor that the final concentrations were only 0.8 to 2.5 ng/*μ*L. In conclusion, genomic DNA from horns of different “Lingyangjiao” species was sufficient to recover whole barcoding sequences of 644 bp, except two powdery horn samples of Saiga antelope, from which only shorter sequences of 349 bp were obtained by nested PCR.

In total, 69 sequences of eight “Lingyangjiao” species were obtained, including 52 from the regular PCR, two from nested PCR, and 15 from GenBank (Table1). Firstly, 67 sequences that were obtained from the regular PCRs and GenBank were trimmed to 644 bp and assembled as data set of full-length barcodes. Alignment of these sequences showed that 459 positions were conserved and the other 185 were polymorphic. The average nucleotide frequencies were 31.1% for T, 25.6% for C, 26.3% for A, and 16.9% for G.

**Figure 2:**
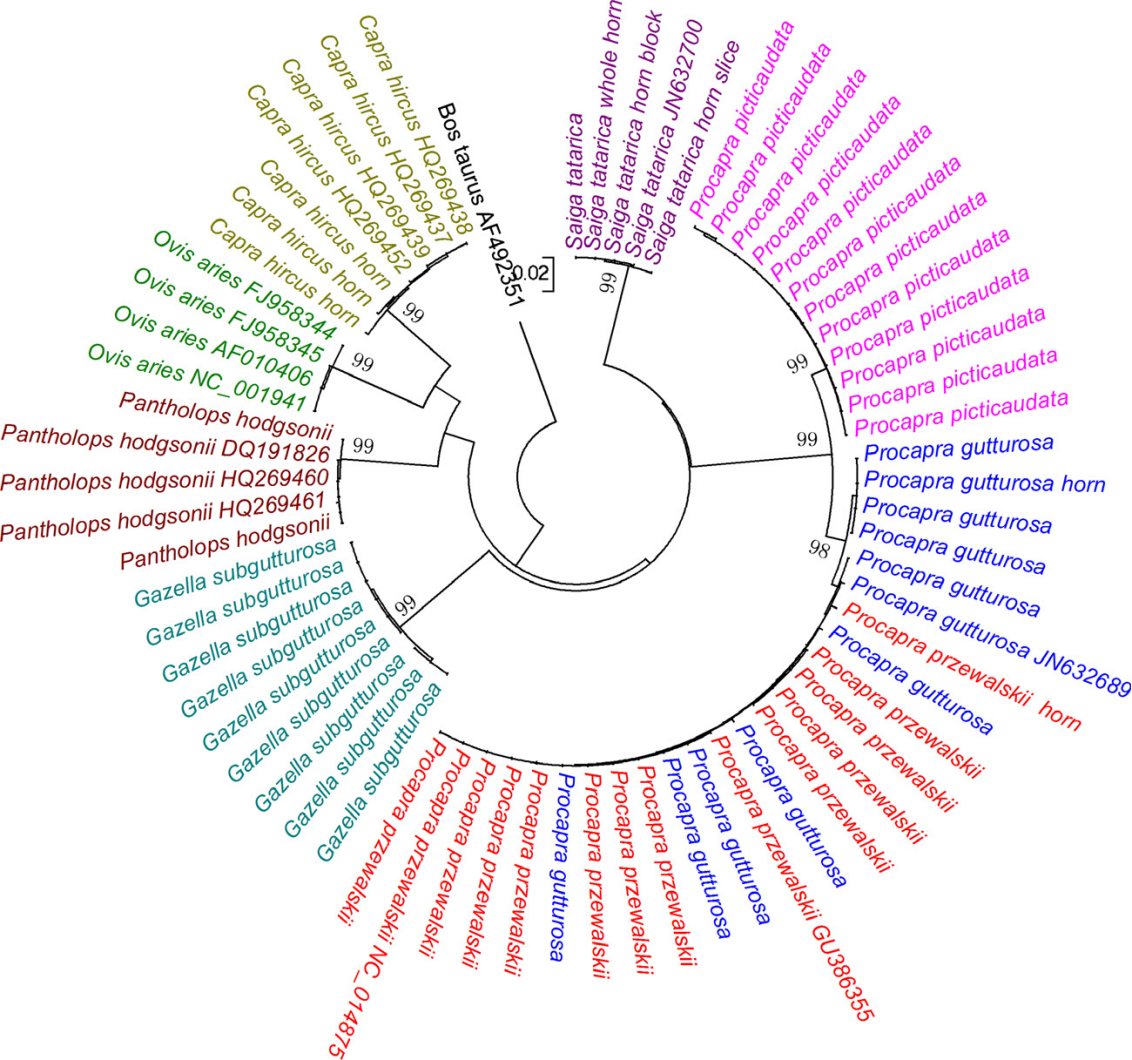
Neighbor-joining (NJ) tree of 644-bp COI sequences of from eight species involved in TCM “Lingyangjiao” markets. The sequences with GenBank accession numbers were retrieved from GenBank. Samples of horns were marked after species names.

Intraspecific and interspecific genetic distances of 67 COI sequences of the full-length barcodes are shown in Table2. Generally, intraspecific K2P distance was low, with an average of 0.24%, whereas interspecific genetic distance averaged at 14.96%. All the species clades in the NJ phylogenetic tree were supported with 99% bootstrap values, except *P. przewalskii* and *P. gutturosa* (Fig.2). High mean interspecific distance can be explained by the fact that the eight “Lingyangjiao” species belonged to six genera and only three were congeneric. Genetic divergences at the family level were proved higher than intrageneric ones in lepidopterans, crustaceans, birds, and bovines (Hebert et al. 2003b, 2004; Lefébure et al. 2006; Cai et al. 2011). Saiga antelope also had high interspecific genetic distances when compared with the other “Lingyangjiao” species (averaged at 15.13%), which makes the identification of Saiga horns more accurate. Advantage was also preserved by *G. subgutturosa*, *P. hodgsonii*, *C. hircus,* and *O. aries*.

**Table 2:** Pairwise genetic distances between and within eight “Lingyangjiao” species based on 644-bp barcodes

Species	[1]	[2]	[3]	[4]	[5]	[6]	[7]	[8]
[1] *Procapra picticaudata*	**0.1**							
[2] *Procapra przewalskii*	2.9	**0.1**						
[3] *Procapra gutturosa*	2.8	0.5	**0.6**					
[4] *Saiga tatarica*	15.1	16.4	15.9	**0.3**				
[5] *Gazella subgutturosa*	16.5	17.3	17.0	13.4	**0.2**			
[6] *Pantholops hodgsonii*	16.8	18.2	17.9	14.6	16.2	**0.1**		
[7] *Ovis aries*	19.9	20.5	20.4	16.0	15.9	11.6	**0.1**	
[8] *Capra hircus*	18.7	19.2	19.4	14.5	16.1	13.9	11.2	**0.4**

The distances were estimated based on Kimura 2-parameter model and showed as percentage. Bold numbers on the diagonal were intraspecific distances, and numbers below the diagram were interspecific distances.

**Figure 3:**
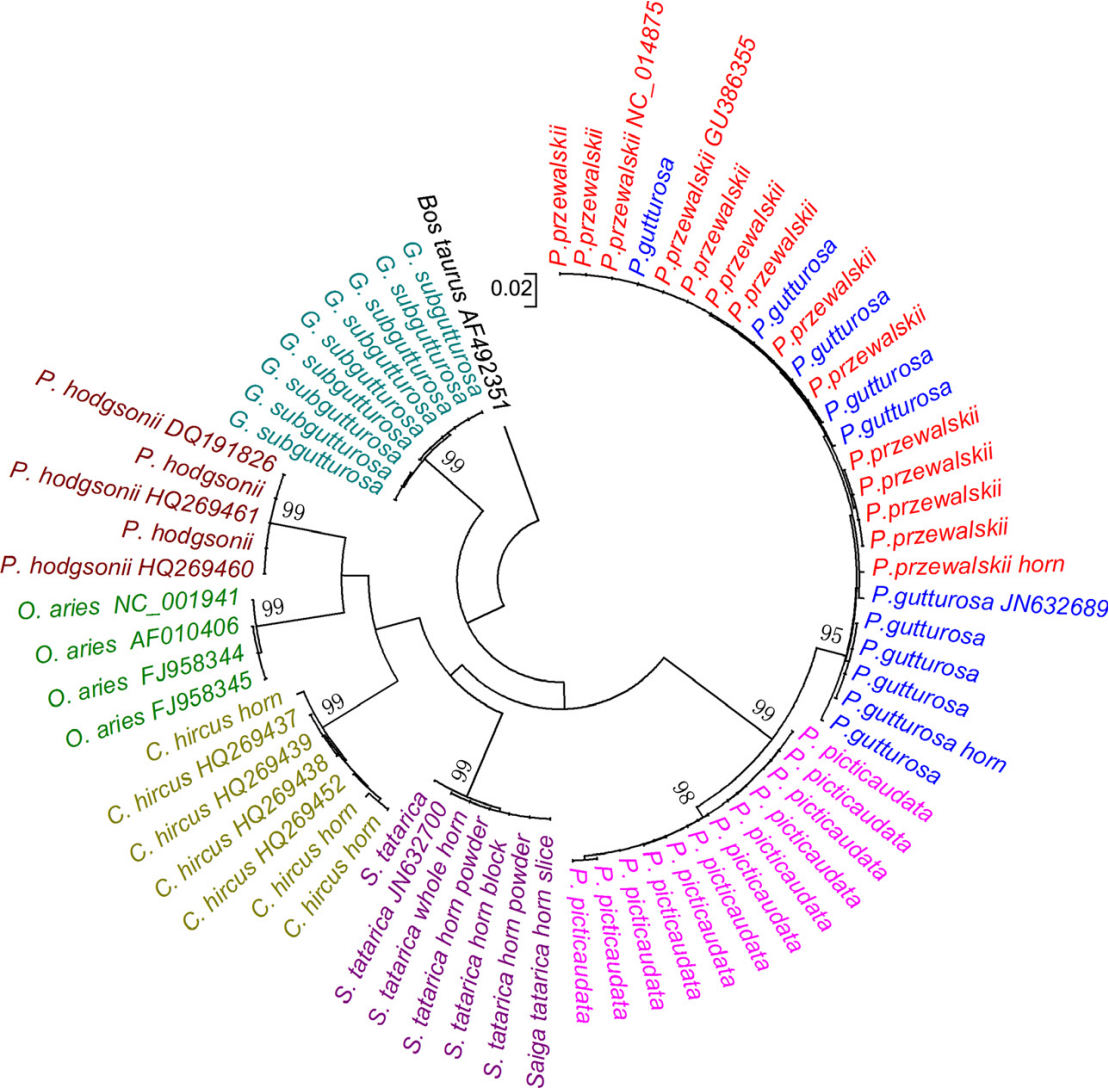
Neighbor-joining tree (NJ) of 349-bp mini-barcodes from eight species involved in TCM “Lingyangjiao” markets. The sequences with GenBank accession numbers were retrieved from GenBank. Samples of horns were marked after species names.

Among the three *Procapra* species, interspecific distance between *P. przewalskii* and *P. gutturosa* was as low as 0.5%, which was overlapped with intraspecific distances of these two species. We retrieved and analyzed the whole mitochondrial genomic sequences of *P. przewalskii* and *P. gutturosa* from GenBank (Accession Numbers: GU386355, NC_014875, and JN632689). The interspecific distance calculated from the 16,548-bp sequences (0.6%) was found to be close to that of COI gene (Table S1). *P. przewalskii* and *P. gutturosa* also formed mixed clade in NJ phylogenetic tree (Figs.2 and 3), making distinction of these two species unclear. *P. przewalskii* and *P. gutturosa* were diverged 0.88 Ma ago, and the short divergence time may account for the low genetic distance (Yang 2011). Besides, the two species lived sympatrically in Inner Mongolia hundreds of years ago, which meant the possibility of genetic introgression (Hu and Jiang 2012).

To investigate the discrimination ability of mini-barcoding sequences from nested PCR, all the 69 sequences were trimmed to 349 bp. Average intra- and interspecific distance of the data set were 0.20% and 15.38%, which were both close to that of 644-bp data set (Table3). All the samples were assigned to the correct species clades with >98% bootstrap values, while *P. przewalskii* and *P. gutturosa* were still indistinguishable (Figs.2 and 3). Similarity between data sets of 644 bp and 349 bp demonstrated that nested PCR system designed in this study was feasible. Validity of mini-barcodes down to 100-bp lengths was also confirmed by studies of other taxa (Bitanyi et al. 2011; Dubey et al. 2011; Zeale et al. 2011). Hence, short barcodes could be an effective alternative for degraded samples from which the full barcodes were hard to recover.

**Table 3:** Pairwise genetic distances between and within eight “Lingyangjiao” species based on 349-bp mini-barcodes

Species	[1]	[2]	[3]	[4]	[5]	[6]	[7]	[8]
[1] *Procapra picticaudata*	**0.1**							
[2] *Procapra przewalskii*	3.1	**0.2**						
[3] *Procapra gutturosa*	3.1	0.5	**0.5**					
[4] *Saiga tatarica*	15.3	17.2	16.8	**0.1**				
[5] *Gazella subgutturosa*	16.0	17.0	16.7	13.9	**0.1**			
[6] *Pantholops hodgsonii*	18.3	20.5	20.4	15.2	16.4	**0.0**		
[7] *Ovis aries*	20.1	22.5	22.5	15.4	16.2	10.2	**0.1**	
[8] *Capra hircus*	18.5	19.9	20.2	14.0	16.1	13.5	11.1	**0.5**

The distances were estimated based on Kimura 2-parameter model and showed as percentage. Bold numbers on diagonal were intraspecific distances, and numbers below the diagram were interspecific distances.

## Conclusions

We developed a COI-based identification protocol to identify horns of Saiga antelope and other “Lingyangjiao” species used in TCM. The protocol covered procedures of extraction of genomic DNA from horn samples, PCR amplification, and analysis of barcoding data sets. Nested PCR system was designed to deal with degraded samples which were sometimes the only available resources in forensic cases. Species diagnosis is a basic requirement of wildlife trade monitoring and laws enforcing in wildlife crimes. The barcoding identification method described here is expected to contribute to monitoring trades of TCM “Lingyangjiao” and support conservation of Saiga antelope and the other endangered ungulates such as *P. picticaudata*, *P. gutturosa*, *P. przewalskii*, *G. subgutturosa*, and *P. hodgsonii*.

## Supporting Information

**Table S1.** Pairwise genetic distances between *P. przewalskii* and *P. gutturosa* based on whole mitochondrial genomic DNA.
