# Supplementary material for: Identification of ungulates used in a traditional Chinese medicine with DNA barcoding technology
Source: Ecol Evol. 2015 Apr 8;5(9):1818–25. doi: 10.1002/ece3.1457 (PMC4485963; doi:10.1002/ece3.1457)
Supplement: Supplementary file 1 [file ece30005-1818-sd1.doc]

**Table S1 Pairwise genetic distances between *P. przewalskii* and *P. gutturosa* based on whole mitochondrial genomic DNA.**

|  | [1] | [2] | [3] | [4] |
| --- | --- | --- | --- | --- |
| [1]*Procapra przewalskii* |  |  |  |  |
| [2]*Procapra gutturosa* | 0.6 |  |  |  |
| [3]*Gazella subgutturosa* | 13.0 | 13.0 |  |  |
| [4]*Saiga tatarica* | 12.9 | 12.8 | 11.8 |  |

The distances were estimated based on Kimura 2-parameter model and shown as percentage.
